# Self-rated work ability as a risk factor for disability retirement

**DOI:** 10.1093/eurpub/ckad121

**Published:** 2023-07-13

**Authors:** Sakari Kainulainen, Marko Elovainio, Mikko Laaksonen, Tuija Jääskeläinen, Harri Rissanen, Seppo Koskinen

**Affiliations:** Diaconia University of Applied Sciences, Helsinki, Finland; Research Program Unit, Faculty of Medicine, University of Helsinki, Helsinki, Finland; Department of Psychology and Logopedics, Faculty of Medicine, University of Helsinki, Helsinki, Finland; Department of Public Health and Welfare, Finnish Institute for Health and Welfare, Helsinki, Finland; Finnish Centre for Pensions, Helsinki, Finland; Department of Public Health and Welfare, Finnish Institute for Health and Welfare, Helsinki, Finland; Department of Public Health and Welfare, Finnish Institute for Health and Welfare, Helsinki, Finland; Department of Public Health and Welfare, Finnish Institute for Health and Welfare, Helsinki, Finland

## Abstract

**Background:**

Simple and efficient survey measures to predict staying in or leaving work are needed. We examined the association of single-item self-rated work ability (SRWA) with disability retirement in two large population-based samples and compared the association of SRWA to two other scales, work ability score (WAS) and self-rated health (SRH), used earlier in studies.

**Methods:**

The study population comprised 6034 participants aged 35–58 from the population-based Health 2000 and FinHealth 2017 cohort studies, pooled together. SRWA, WAS and SRH were all classified in three categories: poor, limited and good. A 36-month follow-up for disability retirement via linkage to electronic records was included in the analysis.

**Results:**

Of the participants, 195 retired during the follow-up. All three measures strongly predicted disability retirement. Hazard ratio (HR) for poor SRWA (vs. good) was 8.48 [95% confidence interval (CI) 5.41–13.28], WAS 7.99 (95% CI 5.62–11.37) and SRH 5.96 (95% CI 4.17–8.51). HR for limited SRWA (vs. good) was 4.35 (95% CI 3.21–5.91), WAS 3.54 (95% CI 2.49–5.04) and SRH 2.27 (95% CI 1.59–3.23). Taking into account gender, age, education and mental health narrowed the gap between poor and limited vs. good work ability as predictors of disability retirement, but the differences remained clear.

**Conclusions:**

Limited or poor self-rated work ability or health are strong predictors of disability retirement. The SRWA measure is a useful survey-measure of work ability in community-based surveys.

## Introduction

The populations in industrialized countries are rapidly ageing, and the dependency ratios are becoming disadvantageous. The improvement of the dependency ratio requires that as many people as possible are fit for work and participate in working life longer than before. Low work ability is a major challenge to labour supply and the sustainability of social protection systems. Promoting the working age population’s ability to work lengthens working careers and contributes as well to improving quality of life of the growing pensioner population.^1^^,^^2^ High-quality measures on work ability are needed to monitor its development and promote sustainable working lives.

Several metrics have been developed to measure work ability (mostly within the working population), but some of the metrics, such as the work ability index (WAI), are relatively complex and not feasible to use in large-scale population surveys.^3^ Much simpler metrics have also been used alongside complex ones. The single-item self-rated health (SRH) is commonly used in psychological research, clinical settings and general population studies, also as an indicator or correlate of work ability.^1^^,^^4^^,^^5^ The subjective assessment of work ability measures more precisely than the overall assessment of health focuses on the balance of job requirements and individual resources.^6^ Previous studies have shown that work ability can be reliably measured with the single-item work ability score (WAS).^3^^,^^7–12^ The simple and concrete self-rated work ability question (SRWA) has also shown its potential^13^^,^^14^ as a general measure of work ability in population surveys. SRWA strongly predicts mortality^14^ and is more strongly associated with future employment than limiting long-standing illness or SRH.^13^ In previous studies, WAS and SRH have been shown to be associated with disability retirement, but the SRWA measure has not been used in such studies.

Compared to the WAS and SRH, the upside of SRWA is that the interpretation of the result is more straightforward. People directly answer whether their working capacity is full or limited or non-existent. Instead, in the WAS and especially in the SRH, both the respondent and the analyser are forced to interpret what the score means. On the other hand, the wider scale in the original WAS brings the possibility of more nuanced analysis and classification of the results. Another possible complicating interpretation of the results is that the WAS compares current working capacity with the best working capacity earlier and in SRH the link to working ability is abstract. If working capacity has always been poor, the current low working capacity measured with WAS is at the same level as it was on the highest (score 10). Therefore, in this study, the ability of SRWA to predict disability retirement in the general population and in selected subgroups of the population will be analysed and compared the results to those obtained with WAS and SRH.

## Methods

### The study population

The participants were from two population-based, nationally representative health examination studies: Health 2000 and FinHealth 2017.^15^ The Health 2000 survey was carried out in 2000 and 2001. The target population consisted of all people aged 18 years or older residing on the Finnish mainland. The sample was drawn from this population with a two-stage stratified cluster sampling procedure.^16^ In total, 8028 Finns over the age of 30 and 1894 aged 18–29 years were invited to participate. In this study, we focus on 35- to 58-year-old participants who were interviewed. The response rate was 87.4% and the number of participants 3722. The material was quite representative and there were no very large differences in non-participation in the different sub-groups. However, men living alone, on low incomes and having been unemployed or studying participated slightly less frequently in the survey.^16^

The FinHealth 2017 health examination study was carried out in 2017 on the random sample of 10 000 persons aged 18 years or older in Finland, using a similar sampling scheme as in the Health 2000 survey. The study consisted of a physical examination and questionnaires. The response rate was 68% and the number of participants 2746 within the age group 35–58 years. Women participated more often than men to FinHealth 2017 study. In foreign language groups, the participation rate was considerably low. Lower participation was also seen among the low educated participants and students or unemployed.^17^

Both surveys were conducted according to the Declaration of Helsinki and they were approved by the Ethics Committees of the Finnish Institute for Health and Welfare and the Helsinki and Uusimaa hospital region, and all participants gave written informed consent. The design, population, and protocol of the individual cohorts have been described in detail elsewhere.^15–17^

Three hundered and thirty-four participants were excluded from the analysis because they were already retired when they took part in the interview or answered the questionnaire. In total, 3430 participants from the Health 2000 study and 2604 from the FinHealth2017 study were included in the analysis. These two representative datasets of 35- to 58-year-old people were combined and the follow-up time was restricted to maximum 36 months. The age limit was set between 35 and 58, so that it was not possible to retire on old-age pension during the monitoring period and it covers people in their middle age. Characteristics of the participants in the pooled dataset are presented in table 1.

**Table 1:** Characteristics of the participants and share of disability retirements during follow-up in the combined data

	*N*	%	# of retired	Retired %	Age when retired
All	6034		195	3.20%	53.2
Sex					
Male	2866	50	94	3.30%	53.5
Female	3168	50	101	3.20%	52.9
Age (years)					
Mean (SD)	46.2	(6.71)			
Education					
Lower	1108	19.2	68	6.10%	53.7
Intermediate	2208	36.7	80	3.60%	53.6
Higher	2712	43.8	47	1.70%	51.7
Mental health problems (GHQ > 3)					
No	4308	81.4	101	2.30%	53.5
Yes	984	18.6	72	7.30%	52.5
Missing info	742	12.3			

### Work ability

Work ability was measured in three ways using a single question. Firstly, work ability was asked with the three-step self-rated work ability question (SRWA): ‘Regardless of whether you are employed or not, please estimate your current work capacity’. Are you ‘completely fit for work’, ‘partially unable to work’ or ‘completely unable to work’? These categories will be referred to as good, limited, and poor work ability, respectively.

The second measure used was an 11-step WAS which is part of the WAI^18^ but is also often used on its own: ‘Assume that your work ability at its best has a value of 10 points. How many points would you give your current work ability?’ The response alternatives vary from 0 to 10. Zero means that one currently cannot work at all and 10 refers to work ability at its best. This 11-step scale was categorized into three categories (0–5 = Poor, 6–7 = Limited, 8–10 = Good).^19^ Classification into four categories was most consistent with the four-step classification of the entire index (WAI),^20^ but we combined categories 3 and 4 into one category to make it more comparable with the three-step work ability measure.

The third measure used was the five-step question on SRH asked with a question: ‘How is your health in general?’ As in previous studies,^21^ this measure was collapsed into three categories: poor or rather poor (poor), average (limited) and rather good or good health (good). All these short measures, SRWA, WAS and SRH are suitable for both currently employed and non-employed persons.

### Disability retirement

Information on disability retirement was obtained from the registers of The Finnish Centre for Pensions until the end of 2020. A disability pension can be granted if one’s work ability has been reduced by at least 40% and the work disability is estimated to last at least 1 year without interruption. Shorter work disability is compensated by sickness allowance. The application must be accompanied by a medical certificate issued by the applicant’s treating doctor. However, the final decision will be taken by insurance physicians, who will consider not only health problems but also social factors such as age, education and work history when making their decision.

### Assessment of potential predictors of disability retirement

Potential predictors of disability retirement included common risk factors for poor work ability: sex, age, educational attainment (Low: primary school; Middle: Vocational or high school; High: College or university) and mental health problems based on the General Health Questionnaire-12 [classified low (0–3) and high (4–12)].^22^

### Statistical methods

We used Cox Regression to estimate hazard ratios (HRs) for the association of work ability with disability retirement as well as to produce survival curves for follow-up period. In Cox regression analysis we used the pooled data. The follow-up for disability retirement lasted from the interview or filling the questionnaire to disability retirement, death or end of follow-up (36 months), whichever came first.

In the primary analyses, we examined whether having limited or poor (reduced) work ability predicted disability retirement with the following steps. First, we assessed the unadjusted associations of the work ability measures with disability retirement, separately for men and women, age groups, educational levels and among those with and without mental health problems. Second, we assessed the associations of poor work ability with disability retirement risk adjusting for education, age, sex and mental health problems. This analysis was done for each indicator of work ability. The risk explained by the predictors was calculated to assess the extent to which the associations of poor work ability with disability retirement were attributable to differences between individuals with reduced work ability and the other individuals on the level of predictors included in the models. To test sensitivity and specificity of the three measures, ROC curve analysis and crosstabulations were used. We conducted all data analyses in SPSS Statistics 27.

## Results

The primary analysis in the combined cohort contained 6034 participants (2866 men, 48% and 3168 women, 52%). The mean age was 46.2 years (SD = 6.7 years). Of the participants, 18.4% had lower, 36.6% intermediate and 45.0% higher educational attainment and 18.6% reported mental health problems (table 1). We identified 195 disability retirements during the follow-up period (3.3% of the participants). According to SRWA, 548 participants (9.2%) were classified as having limited and 67 (1.1%) poor work ability. According to WAS, 742 participants (12.5%) were classified as having limited and 227 (3.8%) poor work ability. Correspondingly, 1310 participants (21.8%) were classified as having limited and 286 (4.7%) poor health. Each of these three measures can be considered to assess work ability, but from different viewpoints. The mutual correlations (Spearman’s rho) between the measures were relatively high (range from 0.42 to 0.59).

Evaluated using SRWA, the unadjusted HR for the risk of disability retirement among those with limited vs. good work ability was 4.35 [95% confidence interval (CI) 3.21–5.91] and 8.48 (95% CI 5.41–13.28) among those with poor vs. good work ability. Unadjusted HRs using WAS were 3.54 (95% CI 2.49–5.04) and 7.99 (95% CI 5.62–11.37) and the corresponding ratios regarding SRH 2.27 (95% CI 1.59–3.23) and 5.96 (95% CI 4.17–8.51), respectively. These associations were evident across sex, education levels, age and among those with and without mental health problems (table 2). However, CIs overlap and show that categories of limited and poor did not differ strongly from each other. The measures used also produced a slightly different result for men than for women as well as people with or without mental health problems when comparing limited and poor work ability. Poor work ability and poor health are stronger predictors of disability retirement than limited work ability or health. This result is visible in both the 2000 and 2017 datasets, but the differences are smaller in the 2017 dataset.

**Table 2:** Association of work ability indicators (SRWA, WAS) and health (SRH) with disability retirement in sub-groups

	Work ability	SRWA	(95% CI)	WAS	(95% CI)	SRH	(95% CI)
2000							
	Limited	3.97	(2.78–5.68)	3.41	(2.27–5.10)	2.18	(1.44–3.31)
	Poor	8.97	(5.32–15.14)	7.42	(4.91–11.21)	6.21	(4.11–9.39)
2017							
	Limited	2.67	(1.42–4.99)	1.94	(0.93–4.06)	1.03	(0.52–2.04)
	Poor	2.87	(1.17–7.04)	4.19	(2.03–8.67)	1.96	(0.96–3.99)
Pooled							
	Limited	4.35	(3.21–5.91)	3.54	(2.49–5.04)	2.27	(1.59–3.23)
	Poor	8.48	(5.41–13.28)	7.99	(5.62–11.37)	5.96	(4.17–8.51)
Sex							
Men	Limited	4.43	(2.82–6.95)	3.26	(1.92–5.54)	1.37	(0.81–2.33)
	Poor	6.57	(3.56–12.13)	8.53	(5.06–14.39)	4.72	(2.88–7.72)
Women	Limited	4.32	(2.85–6.56)	3.85	(2.39–6.20)	3.37	(2.08–5.46)
	Poor	11.56	(5.95–22.48)	7.37	(4.53–11.98)	6.97	(4.14–11.73)
Level of education							
Lower	Limited	3.78	(2.24–6.36)	2.62	(1.41–4.88)	1.63	(0.87–3.06)
	Poor	9.16	(4.40–19.09)	6.23	(3.44–11.28)	3.7	(1.985–6.9)
Intermediate	Limited	4.46	(2.76–7.22)	4.59	(2.68–7.86)	2.83	(1.62–4.95)
	Poor	8.43	(4.14–17.14)	7.73	(4.36–13.72)	7.75	(4.41–13.62)
Higher	Limited	5.5	(2.96–10.23)	3.4	(1.65–7.00)	2.41	(1.22–4.78)
	Poor	7.19	(2.72–19.00)	14.52	(7.24–29.09)	9.34	(4.57–19.08)
Age							
35–44	Limited	7.587	(3.61–15.94)	3.731	(1.53–9.13)	2.369	(1.01–5.58)
	Poor	12.019	(3.95–36.56)	13.43	(5.91–30.52)	9.012	(3.9–20.83)
45–54	Limited	2.946	(1.96–4.44)	2.758	(1.75–4.35)	1.77	(1.11–2.83)
	Poor	6.016	(3.33–10.88)	5.418	(3.37–8.70)	4.304	(2.69–6.89)
55–58	Limited	2.773	(1.50–5.12)	2.297	(1.10–4.79)	0.957	(0.47–1.96)
	Poor	9.728	(3.76–25.19)	4.029	(1.95–8.31)	2.358	(1.12–4.96)
Mental health problems (GHQ > 3)							
No	Limited	3.79	(2.48–5.79)	2.79	(1.74–4.48)	1.48	(0.92–2.37)
	Poor	11.13	(5.64–21.94)	7.87	(4.88–12.70)	5.76	(3.60–9.21)
Yes	Limited	4.38	(2.56–7.49)	4.74	(2.34–9.60)	5.6	(2.48–12.64)
	Poor	5.9	(3.00–11.62)	8.76	(4.42–17.37)	8.86	(3.88–20.20)

*Note*: Cox regression hazard ratios and 95% confidence intervals (95% CI). People with good work ability/health were the reference group, HR = 1.

**Figure 1:**
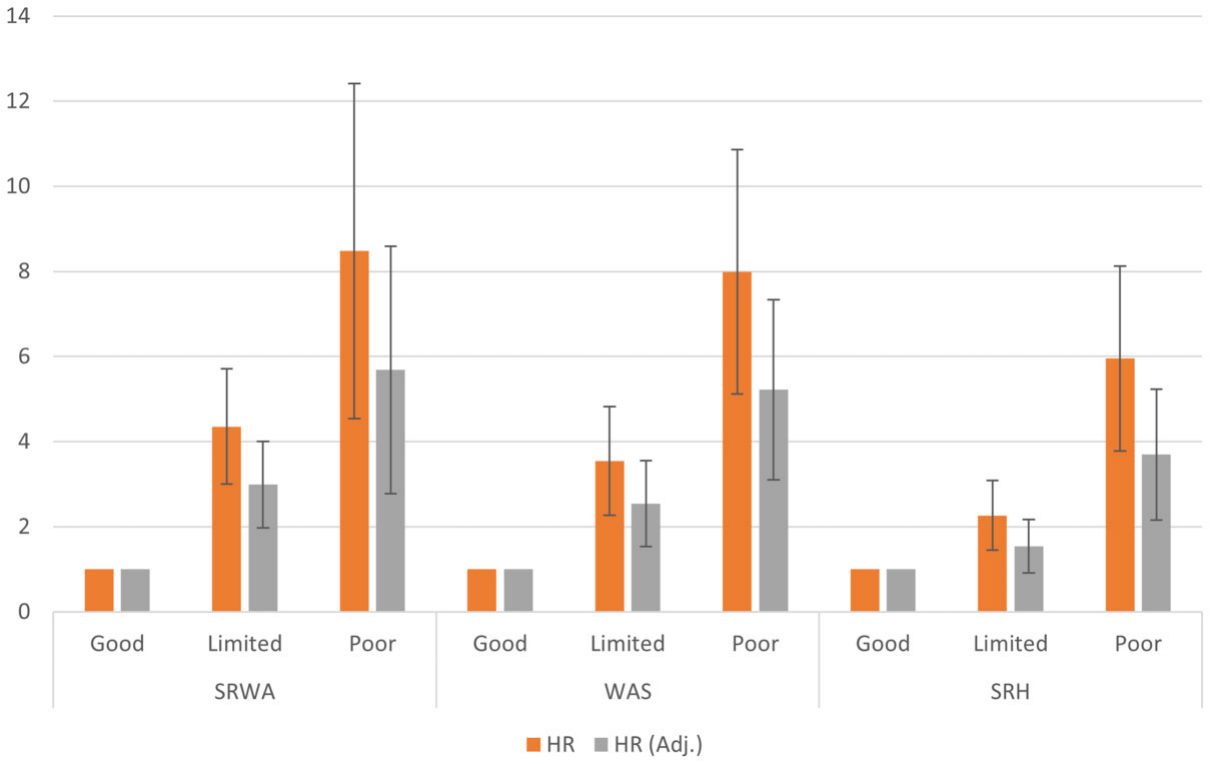
Associations of work ability indicators (SRWA, WAS, SRH) with disability retirement. Crude Cox regression hazard ratios and hazard ratios adjusted by education, age, sex and mental health

When adjusted for sex, age, education and mental health problems, HR for the risk of disability retirement decreased moderately with all measures and in both work ability categories. However, clear differences remained in the risk of disability retirement between those with good work ability compared to those with reduced work ability also after these adjustments (figure 1).

**Figure 2:**
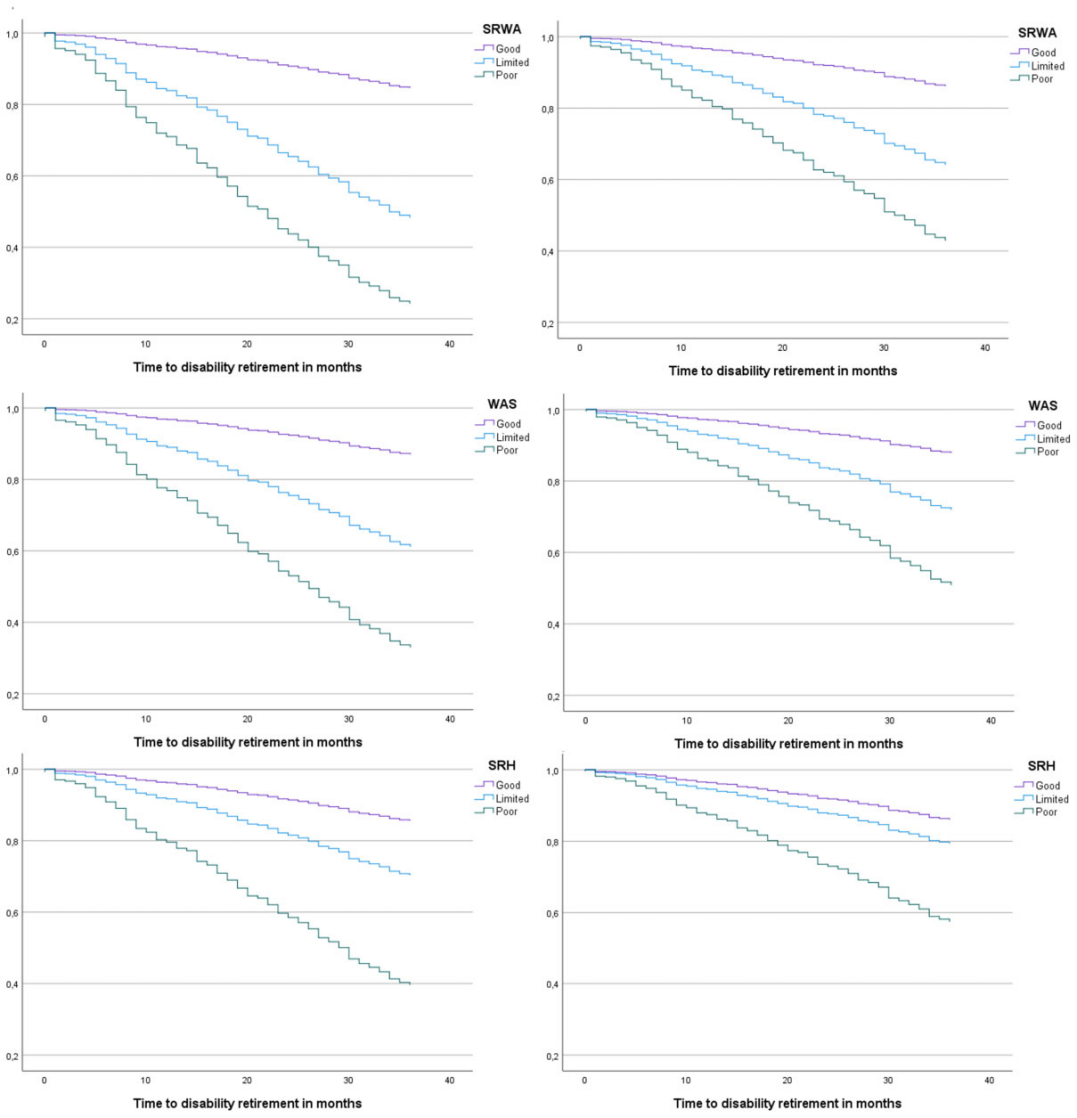
Survival function for SRWA work ability measure without (top left) and with adjustment for education, age, sex and mental health (top right); survival function for WAS (middle left), adjusted (middle right); survival function for SRH (bottom left), adjusted (bottom right)

The ability of all measures to predict disability retirement remained clear throughout the follow-up: reduced work ability clearly predicted disability retirement even when potential confounders or mediators were considered (figure 2).

Self-assessed work ability and health had a clear connection to disability retirement. Depending on the measure, 24–37% of those who assessed their work ability or health as poor were on disability retirement at the end of the follow-up (SRWA 37%, WAS 29%, SRH 24%). In contrast, only less than two per cent of those who assessed their ability to work as good had transferred to disability retirement during the follow-up period (SRWA 1.5%, WAS 1.2%, SRH 1.2%). Of those who had assessed their work ability as limited 15% were on disability retirement at the end of the follow-up when evaluated with SRWA, 9% when evaluated with WAS and 5% when evaluated with SRH. ROC curve (AUC) showed small differences between the measures in sensitivity and specificity (SRWA 0.74, WAS 0.78, SRH 0.77).

## Discussion

The present study using nationally representative samples found that that people with poor self-rated work ability or health are at a higher risk of disability retirement than are people with good work ability. This association was obtained using three single-item measures, all classified into three response categories. The observed around four to eight-fold (SRWA, WAS), and 2- to 6-fold (SRH) excess risk of disability retirement among persons with limited or poor work ability was partly connected with differences in the level of education, and mental health problems between people with reduced work ability and the others. The risk of disability retirement in people with poor work ability was also observed in all subgroups defined by the variables included in the analysis. Furthermore, the SRWA work ability question appeared to have a stronger association with disability retirement than the two other measures.

Although multidimensional measures are valuable when we want to find targets for interventions for enhancing work ability at the individual and organizational level,^23^ in population surveys it is not always possible to use long measures. Thus, short and economic measures with good predictive validity are needed. The SRWA single-item work ability measure seems to be a promising option for such measure capturing the subjective perception of work ability. This measure is simple, easy to understand (easier than the two other measures used) and can be used as an indicator of work ability that is based on the conception people have of their own ability to work.

The present study has important strengths. To the best of our knowledge, with more than 6000 participants from two representative cohorts, the present study is a large and comprehensive examination of the ability of self-assessed work ability to predict future disability retirement. Information on disability retirement was obtained objectively from the nationwide pension register. Some potential limitations of the study should also be considered. Although the response rates in the two cohorts were relatively high, the possibility of selection bias in relation to the investigated exposures and outcome cannot be totally excluded. We could not analyse information on the working conditions of the participants, and we were therefore not able to assess the role of factors related to the demands of work, for example.

The use of pooled data also has its own strengths and weaknesses. The strength was that we were able to increase the number of people on disability pension in this way, which brought more statistical strength to the analyses. However, the 2017 data appeared to differ from the 2000 data in that the share of retirees was lower and perhaps for this reason the predictive capacity of self-assessed indicators decreased clearly and the limited and poor categories converged. However, the basic result remained similar in both datasets, which is why the use of pooled datasets was justified. Our results are expected to be transferable to other countries with a comprehensive disability pension scheme and a similar mechanism for making pension decisions.

## Conclusion

Our findings provide strong population-based evidence that people with reduced self-assessed work ability are at increased risk of disability retirement in the next 3 years. Self-rated work ability (SRWA) is a simple, understandable, and short measure of functional ability with clear reference context, and it is thus potentially useful in surveys which aim to detect persons with reduced work ability or to monitor the time trends and inequalities in work ability or to developing interventions to increase work ability.

## Data Availability

Due to data protection reasons personal data cannot be publicly available. The data controller of the Health 2000 Survey and the FinHealth Study is Finnish Institute for Health and Welfare. Access to confidential data requires permission to handle the data, signed non-disclosure agreement as well as collaboration agreement with Finnish Institute for Health and Welfare (www.thl.fi).
